# Convalescent Homes in the Eastern Counties

**Published:** 1888-02-11

**Authors:** 


### Convalescent Homes in the Eastern Counties.

By Our Roving Correspondent.

With the ever-increasing claims which are made upon the hospitals of the country, and the consequent necessity of discharging patients at the earliest possible moment—to which must be added the fact that the proportion of cures in hospitals is rising with the onward march of surgical and medical science—the need of Convalescent Homes for the less well-to-do of our people is becoming more and more pressing. It is amongst this class that relapses after severe illness are most frequent, for whilst the moderately prosperous man can usually allow himself, or any convalescent member of his family, a lengthy holiday, or, at least, quiet and attention at home, the working and the lower middle-class man can neither afford to move from his not-too-salubrious dwelling, nor, if he stays there, can he be sure of that mental and physical repose which are so essential during the latter stages of recovery. Fortunately, benevolence has stepped in, and very few seaside towns or inland health resorts are now without their Convalescent Homes, where the good work done at the hospital is being continued and perfected. Many of them, it is true, have a hard struggle, but few ever have to close their doors when once they have been opened; and that the day of small things is not to be despised is proved by the existence at this moment of many a beneficent institution whose beginning was of the humblest description. Among the Convalescent Homes on the Eastern Coast there are two which may be chosen for description, because they illustrate what we may call the state of infancy and the condition of complete growth. The one is at Great Yarmouth, and, appropriately enough, is devoted to young folk; the other is at Felixstowe, and receives children of larger growth. It must not be assumed that these are the only Convalescent Homes in the Eastern Counties; there are, on the contrary, flourishing institutions at Lowestoft, Hunstanton, and other places. But these are taken as samples of the whole, and as an incentive to the benevolent to support yet more liberally their counterparts east, west, north, and south.

Within a stone's throw of the beach, and exposed on one of its sides to the full force of the stormy winds that not unfrequently come tearing across the German Ocean, the visitor to Yarmouth will observe a fair-sized house, having over its door the inscription "Eastern Counties Children's Convalescent Home." If his walks do not lead him to that particular spot, he will have noticed on the sands and in the streets small parties of merry children, whose bright cloaks suggest the Red Ridinghood of fairy lore, and afford a marked contrast to the sombre dress of the young woman who attends them. The little ones range in age from the school-girl of thirteen and the frolicsome boy of ten to the tiniest toddling specimen of humanity. Nay, even the non-toddlers are not beneath the attention of the Home, which possesses three perambulators, which in the summer time are seldom without their full complement. It should be explained here that the inscription above quoted is a misnomer, and is to be replaced by another board in which the geographical limitation will be omitted; for the little patients come from all parts of the kingdom, London sending a very considerable number in the course of each year. The Home, however, still deserves its old title in another sense. It had its origin in Norwich, and although the circle of its benefactors is ever widening, still a sensible proportion of the subscribers are residents in that ancient city and its neighbourhood. It benefits, too, by the Hospital Saturday and Sunday collections in the county town.

The history of the Home is a brief one, but encouraging. It originated less than five years ago by the devotion of a little cottage at Cromer to the reception of sick children from Norwich. But that was a somewhat out-of-the-way place—more so then than now—and a move was made to Great Yarmouth. First one house and then another was bought, until a whole block—with the exception of six or seven rooms—has been acquired. Only last year an extension was made which provided the little inmates with that inestimable boon, a play-room for wet weather; and upon the remaining apartments in the building the management has already cast greedy eyes. Let us hope that before next season comes round these also will be added, to the lasting benefit of generations of tiny patients.

So much for the history of the Home. Now for the Home itself. On the day on which my visit was made, the weather was dull but dry, with a more than slight breeze from the north, which made a long line of surf on the treacherous sand banks a mile or so out at sea. The matron, Miss Ellen J. Turner, was absent on her holiday, but her place as my guide was cheerfully taken by Miss Lucy Hansell, a member of a Norwich family which has given much, both of time and of money, to hospital work in the county. The inspection did not take long, for of the twenty-one inmates—there were thirty-five a month or two before in the height of the "season"—only three were at home, the rest being out for their afternoon walk. Of these three, one had just been admitted from Norwich, recovering from treatment in the hospital there for a disease of the hip-joint; and the other two were spending their last day at Yarmouth, after a month's stay. The contrast between them was very striking, for the home-going couple looked as if the days of their convalescence had long passed—even if they had ever been ill at all, which, if one had met them anywhere else would have seemed doubtful—whilst the Norwich girl evidently wanted a lot of building up. I spoke to the youngest of the two former—a brown faced, chubby lass of seven or eight who hailed from London—but a guarded reference to the journey of the morrow was so productive of signs of sorrow that I hastened to change the subject. The little one would probably be pleased enough to see her parents once more, but the free, healthy life of the past month was too enjoyable to be relinquished without a pang. "They mostly leave with regret," said my guide. "I saw three off to London yesterday." And when we reached the matron's-room again after our tour, there was a letter from the mother of one of them which announced the safe arrival of "Harold," and continued: "I beg to thank you, Miss Turner, and the nurses for your kind care of him. He has greatly improved. We do not expect him to grow fat while he grows so tall. I am sure he enjoyed his stay at the Home, for he is full of it, and the nice things he had there, particularly the buns and the 'lovely' meat dumplings." From this letter it is evident that the savoury morsel which is associated by name with the county of Norfolk is not banished from the dietary of the convalescent home.

*(To be continued.)*

